# Screening on binary Ti alloy with excellent mechanical property and castability for dental prosthesis application

**DOI:** 10.1038/srep37428

**Published:** 2016-11-22

**Authors:** H. F. Li, K. J. Qiu, W. Yuan, F. Y. Zhou, B. L. Wang, L. Li, Y. F. Zheng, Y. H. Liu

**Affiliations:** 1 Department of Materials Science and Engineering, College of Engineering, Peking University, Beijing 100871, China; 2 Musculoskeletal Research Laboratory, Department of Orthopaedics & Traumatology, The Chinese University of Hong Kong, Shatin, Hong Kong, China; 3 Center for Biomedical Materials and Engineering, Harbin Engineering University, Harbin 150001, China; 4 General Dental Department, School of Stomatology, Peking University, Beijing 100081, China

## Abstract

In the present study, the microstructure, mechanical property, castability, corrosion behavior and *in vitro* cytocompatibility of binary Ti–2X alloys with various alloying elements, including Ag, Bi, Ga, Ge, Hf, In, Mo, Nb, Sn and Zr, were systematically investigated, in order to assess their potential applications in dental field. The experimental results showed that all binary Ti‒2X alloys consisted entirely *α*–Ti phase. The tensile strength and microhardness of Ti were improved by adding alloying elements. The castability of Ti was significantly improved by separately adding 2 wt.% Bi, Ga, Hf, Mo, Nb, Sn and Zr. The corrosion resistance of Ti in both normal artificial saliva solution (AS) and extreme artificial saliva solution (ASFL, AS with 0.2 wt.% NaF and 0.3 wt.% lactic acid) has been improved by separately adding alloying elements. In addition, the extracts of studied Ti‒2X alloys produced no significant deleterious effect to both fibroblasts L929 cells and osteoblast-like MG63 cells, indicating a good *in vitro* cytocompatibility, at the same level as pure Ti. The combination of enhanced mechanical properties, castability, corrosion behavior, and *in vitro* cytocompatibility make the developed Ti‒2X alloys have great potential for future stomatological applications.

Biomedical metallic materials used in the clinical dentistry include Au-based alloys, Ag-based alloys, Pd-based alloys, Co‒Cr alloys, Ni‒Cr alloys and Ni-Cr-Be alloys^1^,^2^,^3^. Among these clinical used dental alloys, noble alloys, including Au-based, Ag-based and Pd-based alloys, have the major fatal defect of high cost. On the other hand, the base metal alloys, such as Co-Cr alloys, Ni-Cr alloys and Ni-Cr-Be alloys, there are great concerns about their biological safety following numerous reports of the toxicity of nickel and beryllium. Nickel is considered one of the most common causes of allergic dermatitis and is responsible for more allergic reactions than all other metals combined. Beryllium, present in many alternative alloys, improves castability of Ni-Cr alloys by forming a low melting point of eutectic Ni-Be constituent. Unfortunately, beryllium is considered a potential carcinogen, presenting a problem for dental laboratory technicians because beryllium is released during casting and finishing procedures^4^,^5^.

In recent years, titanium has become a material of great interest in prosthodontics. Commercially pure (CP) titanium and its alloys are alternatives to gold and base metal alloys.

CP titanium offers excellent biocompatibility, good corrosion resistance at room temperature, and characteristics such as low density and high mechanical resistance. In theory, the light weight of titanium and its high strength-to-weight ratio permit the design of more functional and comfortable prostheses^6^,^7^. Pure Ti is the most biocompatible metal available for dental casting; however, there is great concern about its quite poor castability since it could result in marginal failures in the restoration, leading to mis-adjustment of the prosthetic parts^8^. In spite of the many desirable characteristics of titanium, there are casting difficulties related to the high melting point of titanium and extreme chemical reactivity at elevated temperatures^9^,^10^.

There have been investigated and developed a great number of new binary Ti alloys over the last two decades^11^,^12^,^13^,^14^,^15^,^16^,^17^,^18^,^19^,^20^,^21^,^22^. Among the various alloying elements of binary Ti alloys, precious metals such as Au, Ag, Pd and Pt are mainly for improving the corrosion resistance of the alloys^11^,^12^,^13^. High-melting-point rare metals such as Mo, Nb, Hf, Zr and Ta are used to enhance the strength and wear resistance of Ti alloys^14^,^15^,^16^,^17^. The fusible metals such as Sn, Ge, In, Ga and Bi are focused on reducing the casting difficulty of Ti alloys^18^,^19^,^20^,^21^,^22^. Additionally, and importantly, all of these alloying elements are considered to have good biocompatibility. However, as the eligible dental materials, the alloys are expected to have adequate mechanical property, good castability, superior corrosion resistance and excellent biocompatibility simultaneously.

In the present work, ten alloying elements (Ag, Bi, Ga, Ge, Hf, In, Mo, Nb, Sn and Zr) were selected and added into binary Ti alloys. The castability of these alloys would be emphasized for evaluating their feasibility as dental prostheses. The Ti‒2X alloys were prepared and studied in order to screen one or several optimum alloy elements for dental Ti alloys with sufficient mechanical property, improved castability, excellent corrosion resistance and outstanding biocompatibility. And the present work would provide a directly useful guideline on the further study of Ti-based alloys for potential dental applications.

## Results

### Microstructures properties of Ti-based alloys

**Figure 1:**
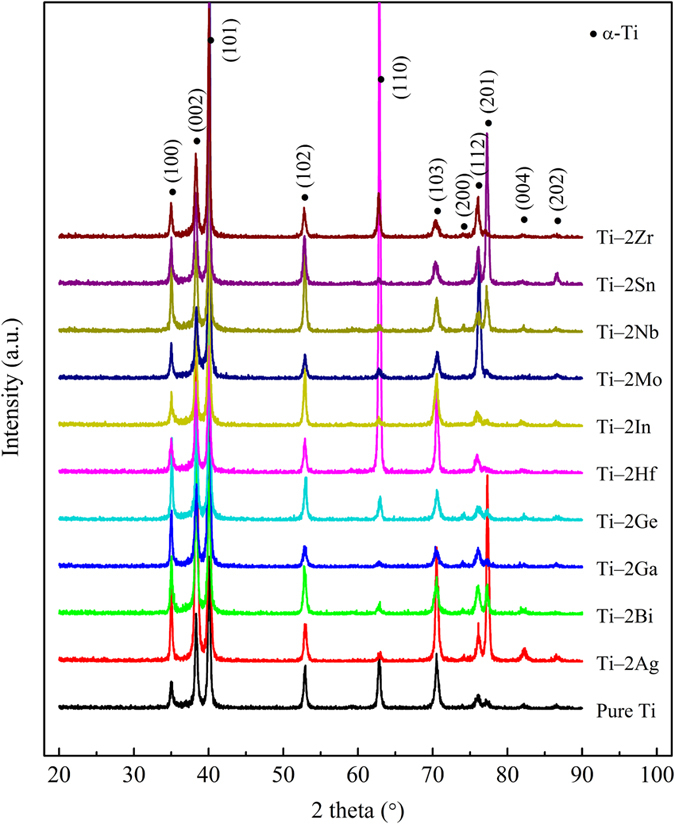
X-ray diffraction patterns of as-cast pure Ti and Ti‒2X alloys at room temperature.

**Figure 2:**
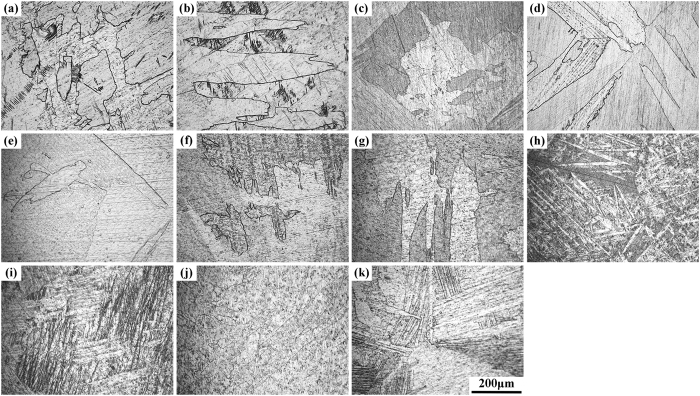
Optical micrographs of as-cast pure Ti and Ti‒2X alloy samples: (**a**) pure Ti, (**b**) Ti‒2Ag, (**c**) Ti‒2Bi, (**d**) Ti‒2Ga, (**e**) Ti‒2Ge, (**f**) Ti‒2Hf, (**g**) Ti‒2In, (**h**) Ti‒2Mo, (**i**) Ti‒2 Nb, (**j**) Ti‒2Sn and (**k**) Ti‒2Zr alloys.

Figure 1 demonstrates the XRD patterns of pure Ti and Ti‒2X alloys at room temperature. It can be noticed that all the Ti‒2X alloys, as well as pure Ti, exhibited a single α phase with hexagonal close packed (hcp) crystal structure. However, in comparison with pure Ti, the intensity of the α phase peaks in the various Ti‒2X alloys behaved differently with the addition of different alloying elements. The optical micrographs of the above mentioned pure Ti and Ti‒2X alloys are shown in Fig. 2. It is obvious that pure Ti exhibited a typical as-cast microstructure with serrated, irregular grain boundaries, indicating a rapid cooling happened during the casting process. The microstructure of Ti‒2X alloys were mainly manifested as columnar grains and/or equiaxial grains, which depending on the different kinds of alloying elements added. To be specific, as shown in Fig. 2(b,d and e), for Ti‒2Ag, Ti‒2Ga and Ti‒2Ge alloys, respectively, the columnar grains were coarser and the grain boundaries became more regular than pure Ti. However, as illustrated in Fig. 2(h,i and k), for Ti‒2Mo, Ti‒2 Nb and Ti‒2Zr alloys, respectively, the fine acicular structures were appeared in the interior of the grains. On the other hand, while adding elements of Bi, Hf, In and Sn in to pure Ti, i.e. for the Ti‒2Bi, Ti‒2Hf and Ti‒2In alloys, there were no apparent changes in the microstructure when compared to pure Ti.

### Mechanical properties of Ti-based alloys

**Figure 3:**
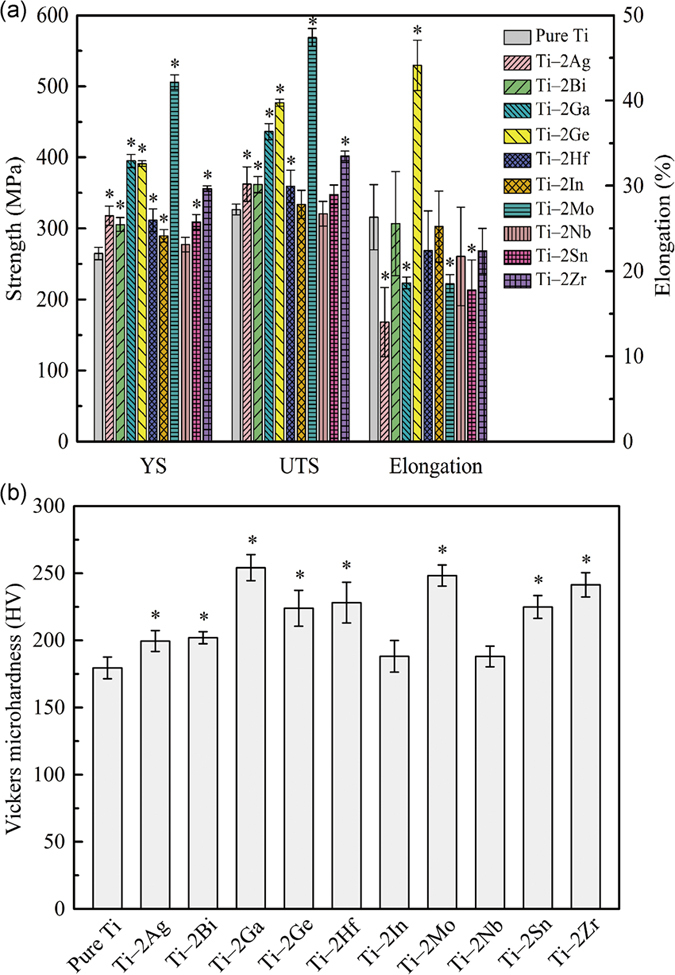
Tensile properties (**a**) and Vickers microhardness (**b**) of as-cast pure Ti and Ti‒2X alloys at room temperature.

Figure 3(a) shows the tensile properties of as-cast pure Ti and Ti‒2X alloys at room temperature. It can be seen that, both the Yield Strength (YS) and Ultimate Tensile Strength (UTS) of the Ti‒2X alloys are much higher than that of pure Ti. The YS and the UTS of pure Ti are only 260 MPa and 325 MPa respectively. However, after adding alloying elements, The YS and the UTS of the Ti‒2X alloys have enhanced up to 500 MPa and 570 MPa respectively. The pure Ti and Ti‒2X alloys exhibited high tensile elongations, and all of them are above 15%, with the highest value of 45% (Ti‒2Ge). Figure 3(b) showed the microhardness of pure Ti and Ti‒2X alloys. It can be seen that after adding the alloying elements, the microhardness of Ti can be enhanced obviously. The microhardness value of pure Ti is only 178 HV, while the microhardness of Ti‒2X alloys are increased up to 254 HV.

### Castability of Ti-based alloys

**Figure 4:**
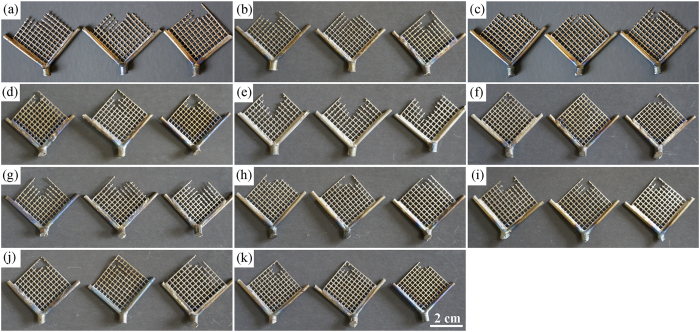
Casting properties of as-cast pure Ti and Ti‒2X alloy samples: (**a**) pure Ti, (**b**) Ti‒2Ag, (**c**) Ti‒2Bi, (**d**) Ti‒2Ga, (**e**) Ti‒2Ge, (**f**) Ti‒2Hf, (**g**) Ti‒2In, (**h**) Ti‒2Mo, (**i**) Ti‒2 Nb, (**j**) Ti‒2Sn and (**k**) Ti‒2Zr alloys.

Figure 4 shows the casting images of as-cast Ti‒2X alloy specimens, with pure Ti as control. According to the casting images, the castability values of Ti‒2X alloys were calculated. It can be observed and calculated that, after adding alloying elements, most of the castability of Ti‒2X alloys has been significantly enhanced when compared with pure Ti (*p* < 0.05), except for the Ag, Ge and In alloying groups. To be specific, Bi, Ga, Hf, Mo, Nb, Sn and Zr increased the castability value by 11.7%, 14.2%, 19.8%, 12.3%, 10.0%, 15.1% and 14.6%, respectively.

### Electrochemical corrosion behaviors of Ti-based alloys

**Figure 5:**
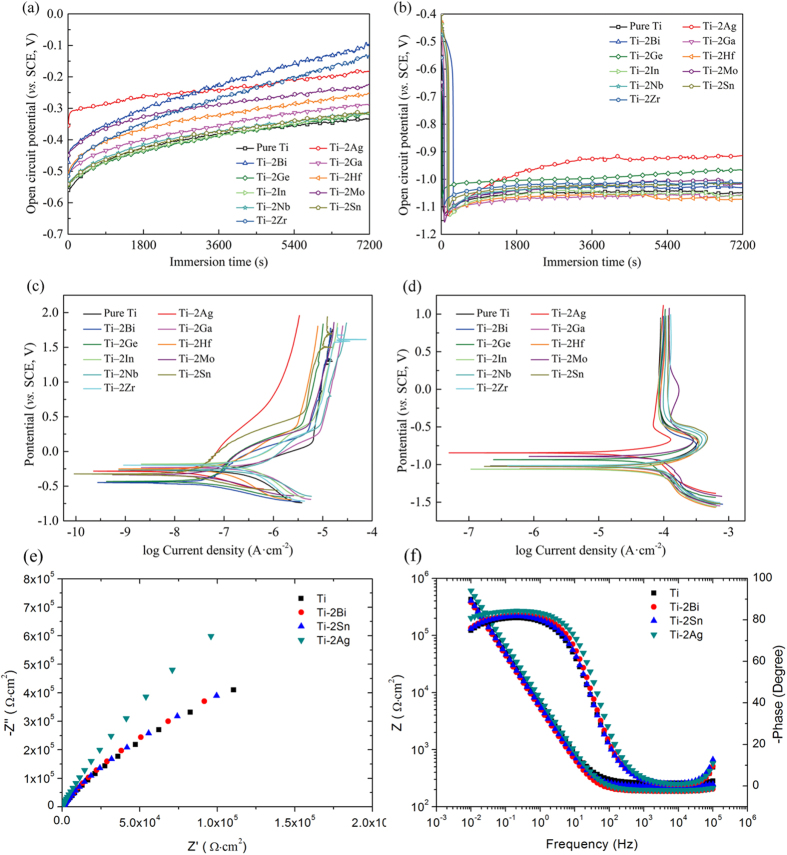
Open circuit potential curves (**a** and **b**), potentiodynamic polarization curves (**c** and **d**) and EIS analysis ((e) Nyquist plots and (f)Bode plots) of as-cast pure Ti and Ti‒2X alloys in normal artificial saliva solution (AS) (**a**,**c**), (**e** and **f**) and extreme artificial saliva solution with 0.2% NaF and 0.3% lactic acid (ASFL) (**b** and **d**).

Figure 5(a and b) showed the OCP variation of as-cast pure Ti and Ti‒2X alloys with immersion time in both AS (Fig. 5(a)) and ASFL(Fig. 5(b)) solutions. It can be seen that the changing trends of OCPs for Ti‒2X alloys are distinctly different for samples tested in the AS and ASFL solutions. In AS solution, the OCP curves of all Ti‒2X alloys took on an upward trend and became stable gradually. On the other hand, when tested in the ASFL solution, the OCP curves of all Ti‒2X alloys as well as pure Ti declined sharply at the initial stage and then remained stable at a lower potential. The average OCP values (after 2 h immersion) of as-cast pure Ti and Ti‒2X alloys are listed in S1. From S1, we can get the information that the OCP values of Ti‒2X alloys in both the AS solution and the ASFL solution are higher than that of the pure Ti, indicating enhanced corrosion resistance after adding alloying elements. Potentiodynamic polarization curves of as-cast pure Ti and Ti–2X alloys in both AS and ASFL solutions are shown in Fig. 5(c and d). Corrosion parameters (*E*_corr_ and *I*_corr_) calculated from the polarization curves are listed in S1 as well. It can be seen that the passivation current densities and corrosion current densities of Ti‒2X alloys in both the AS solution and the ASFL solution are lower than that of pure Ti, which further conforms the enhanced corrosion resistance after adding alloying elements. Among the various alloying elements, Ag, Bi, Ge, Mo and Sn showed the most enhanced corrosion resistance among other alloying elements. In order to further understand the influence of the alloying elements to the corrosion behavior and the characteristic of the passive layers, we performed the electrochemical impedance spectrum (EIS) analysis and X-ray photoelectron spectroscopy (XPS) analysis. Figure 5(e and f) demonstrate the Nyquist plots(e) and Bode plots(f) of pure Ti and Ti‒2X alloys. It can be noticed that all the Nyquist plots in the impedance spectra are characterized by a large depressed semicircle and the diameter of the semicircle becomes larger with the addition of alloying elements, which indicates a nobler electrochemical behavior for the Ti‒2X alloys compared with pure Ti. From the Bode magnitude plots shown in Fig. 5(f), it can be seen that in the high frequency (100–10^5^ Hz), a flat portion of curves (the slope is about 0) is observed due to the response of electrolyte. In the low frequency ranges (0.01–100 Hz), the impedance spectrum displays a linear slope of about −1, which is the characteristic response of a capacitive behavior of passive film. There are two characteristic regions in the Bode phase plots in Fig. 5(f). In the high frequency range, the phase angle drops to 0 degree with the response of electrolyte resistance; in the low frequency, the phase angle remains near to −80 degree indicating a typical passive film presented on the surface and a near capacitive response for passive film. It should be noted that the maximum phase angle increase with the addition of alloying elements, which indicates a nobler corrosion behavior of Ti‒2X alloys. The addition of alloying elements is helpful to increase the resistance of the passive film for pure Ti. The Rs(QpRp) model (Supplementary information S3), was used as the equivalent circuit model to fit the EIS data in the case of passive film. Values of the circuit parameters obtained using the equivalent electrical circuit Rs(QpRp) are listed in Supplementary information S4. As shown in S4, the higher Rp and lower Qp of the Ti‒2X alloys indicate the Ti‒2X alloys have a nobler electrochemical corrosion behavior compared to pure Ti. The XPS analysis results are shown in Supplementary information S5. It can be seen in S5 that besides TiO_2_ (the passive film composition of pure Ti), the passive film formed on the surface of Ti‒2X alloys contains the oxide of the alloying elements (such as AgO for Ti‒2Ag, Bi_2_O_3_ for Ti‒2Bi illustrated in Supplementary information S5).

### Evaluation of *in-vitro* cytocompatibility of Ti-based alloys

**Figure 6:**
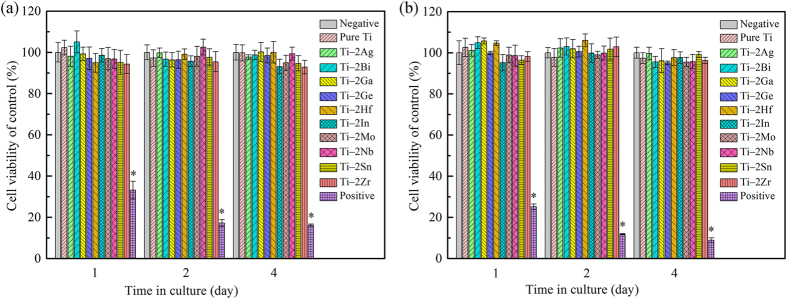
Cell viability of (**a**) L929 and (**b**) MG63 cells cultured in extracts of as-cast pure Ti and Ti‒2X alloys for 1, 2 and 4 days respectively.

**Figure 7:**
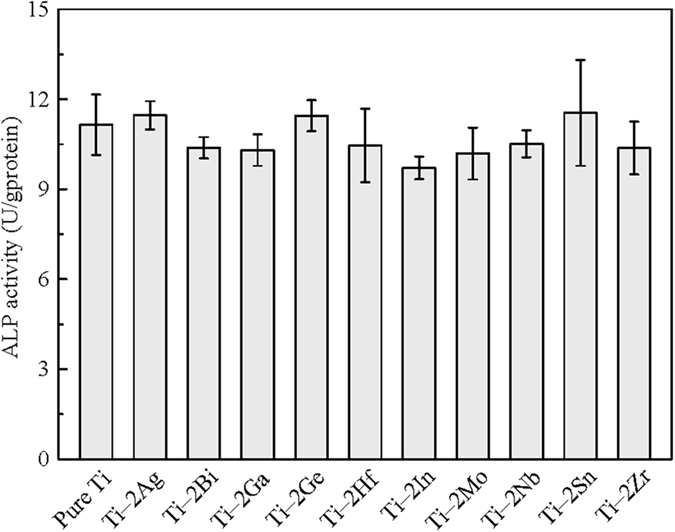
ALP activity of MG63 cells cultured in extracts of as-cast pure Ti and Ti–2X alloys for 7 days.

Figure 6 shows the relative viability of L929 and MG63 cells cultured in extracts of as-cast pure Ti and Ti–2X alloys for 1, 2 and 4 days. It could be seen that, after each culture period, the cell viabilities of both L929 and MG63 cells cultured in the extract of pure Ti were almost the same as in cell culture medium (negative group), representing a good cytocompatibility feature. After 4 days of culture, the extracts of all Ti‒2X alloys exhibited high cell viabilities to both L929 and MG63 cells, the cell viabilities are over 93% and 95%, respectively. The statistical analysis indicated no significant difference among Ti–2X alloys, cell culture control group and pure Ti (p > 0.05). Figure 7 showed the ALP activity of MG63 cells cultured in extracts of as-cast pure Ti and Ti–2X alloys for 7 days. It can be seen from Fig. 7 that there is no significant difference between pure Ti and Ti–2X alloys (p > 0.05). Similar with the MTT assay, it can be inferred that Ti–2X alloys did not inhibit the proliferation of both L929 and MG63 cells and differentiation of osteoblast-like MG63 cells, thereby showing excellent *in vitro* cytocompatibility.

## Discussion

### Mechanical properties of Ti-based alloys

Generally speaking, mechanical properties of casting alloys mainly depend on their microstructures and phase compositions, which are determined by the kind and amount of alloying elements added. It can be noticed from Fig. 1 that the addition of 2 wt.% alloying elements did not change the phase constitution of Ti. Therefore, the improved mechanical properties of Ti‒2X alloys are largely derived from solid solution strengthening of the alloying elements atoms in α‒Ti phase. Due to different alloying elements with different solubility and atomic radius, the solid solution strengthening effects are also different, as seen in Fig. 3(a). Mo has the maximal strengthening effect to Ti, which the UTS of Ti‒2Mo alloy reached up to 570 MPa, almost two times as much as that of the pure Ti.

### Castability of Ti-based alloys

**Figure 8:**
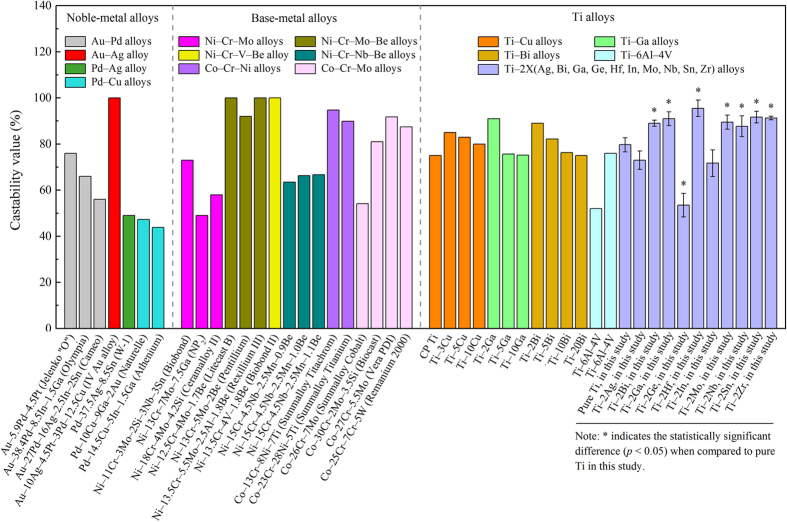
Comparison of the castability of various dental alloys.

Among the various properties of the alloys used in stomatological applications, castability deserves more attention since it is of great clinical interest, as low castability could result in marginal failures in the restoration, leading to mis-adjustment of the prosthetic parts^23^. Figure 8 compares the castability of the various dental alloys^5^,^21^,^22^,^24^,^25^,^26^,^27^,^28^,^29^. It is quite clear that, except for the Ag, Ge and In alloying groups, all the other alloying elements, including Bi, Ga, Hf, Mo, Nb, Sn and Zr, demonstrate significantly increased castability compared with pure Ti and the clinical widely used Au-based, Pd-based and Ni-Cr dental materials. The excellent castability of the present developed Ti‒2X would guarantee a much easier process for the applications in stomatology.

### Corrosion resistance and cytocompatibility of the Ti-based alloys

The corrosion resistance of dental alloys in the oral environments is quite crucial to the longevity of the stomatological biomaterials. The passivation current densities and corrosion current densities of Ti‒2X alloys in both the AS solution and the ASFL solution are lower than that of pure Ti, which means the enhanced corrosion resistance after adding alloying elements. The excellent corrosion resistance of the Ti‒2X alloys would guarantee the long term use of the materials in the oral environments. The present *in vitro* cytocompatibility results showed that Ti‒2X alloys exhibited the good cytocompatibility for L929 fibroblast cells and MG63 osteosarcoma cells after 4 days’ culture. The statistical analysis results showed that there is no significant difference between these Ti‒2X alloys and pure Ti. The alloying elements Hf, Mo, Nb, Sn and Zr which used to add into Ti alloys have long been considered low cytotoxic metals^30^. Ag and Ga has a long history of use in dental alloys, though the Ag^+^ and Ga^3+^ were reported to be relatively high cytotoxicity *in vitro*^31^, the addition of 2 wt.% would not affect the good cytocompatibility of Ti, which was also proved in present study. Actually, Chandler *et al*.^32^ evaluated the effect of Ga^3+^ and In^3+^ and their concentration (0.001~1.0 mmol/L) on L929 cells. The results demonstrated that Ga and In ions have no significant cytotoxicity. In addition, investigation of metal cations cytotoxicity used in dental cast alloys showed that the TC_50_ (toxic concentration by which 50% of the cells in a culture are killed) of In^3+^ and Ga^3+^ for L929 cells was 2310 μM (263.23 mg/L) and 1530 μM (106.67 mg/L), respectively, which were much higher than that of other toxic metal ions such as Zn^2+^, Hg^2+^ and Cd^2+ ^33. Bi is also recognized as a less toxic heavy metal element and has minimum threat to the environment, and the medicinal application of Bi compounds has a history of over 250 years^34^,^35^. In our previous study^22^, it was also proved that the Ti‒Bi alloys produced no significant deleterious effect to L929 cells and MG63 cells. Besides, Ge was also reported to be considered an element of rather low risk to human^36^. From the above, and combined with the cytotoxicity results of present study, the Ti‒2X alloys exhibited an excellent *in vitro* cytocompatibility. However, the current work is based on the limited *in vitro* study, further animal studies and pre-clinical studies are needed to confirm the long term biocompatibility of the above mentioned Ti‒2X alloys.

A series of binary Ti‒2X alloys (X = Ag, Bi, Ga, Ge, Hf, In, Mo, Nb, Sn and Zr) were prepared and their microstructures, mechanical properties, castability, corrosion behaviors and *in vitro* cytocompatibility were investigated to evaluate their feasibility as potential dental materials and the effects of alloying element additions on the properties of Ti were also discussed. The following conclusions can be drawn.

1. All Ti‒2X alloys consisted entirely *α*–Ti phase with hexagonal close packed (hcp) crystal structure and showed typical columnar grains at room temperature.
2. All Ti‒2X alloys showed significantly improved strength and microhardness than that of pure Ti.
3. The castability of pure Ti has been significantly improved by adding alloying elements Bi, Ga, Hf, Mo, Nb, Sn and Zr.
4. The passivation current densities and corrosion current densities of Ti‒2X alloys in both the AS solution and the ASFL solution are lower than that of pure Ti, which means the enhanced corrosion resistance after adding alloying elements.
5. The extracts of studied Ti‒2X alloys produced no significant deleterious effect to both fibroblasts L929 cells and osteoblast-like MG63 cells, indicating a good *in vitro* cytocompatibility, at the same level as pure Ti.

To sum up, adding a minor amount (2 wt.%) of alloying elements, especially for Bi, Ga, Hf and Mo, has improved the mechanical properties, castability, corrosion behavior, and *in vitro* cytocompatibility of Ti comprehensively, which indicated these alloys (Ti‒2Bi, Ti‒2Ga, Ti‒2Hf and Ti‒2Mo) have a great potential for stomatological applications.

## Methods

### Alloys preparation

The as-cast binary Ti‒2 wt.% X alloys with various alloying elements (Ag, Bi, Ga, Ge, Hf, In, Mo, Nb, Sn and Zr) were prepared from sponge Ti (99.5% in purity) and respective high-purity metals (99.9% in purity) in a non-consumable arc melting furnace under an Ar atmosphere. Each ingot was re-melted six times by inversion to improve its chemical homogeneity. The chemical compositions of prepared Ti‒2X alloys were detected by energy dispersive spectrometry (EDS, Hitachi S-4800 SEM, Japan) and the results are given in S2. Different specimens were cut by electro-discharge machining for various tests.

### Microstructural characterization

The microstructure of as-cast binary Ti‒2X alloys was examined using an optical microscope (OM, Olympus BX51 M, Japan), after being polished and etched via a standard metallographic procedure. The etching solution is a mixture of HF, HNO_3_ and H_2_O (5%:15%:80% in volume). An X-ray diffractometer (XRD, Rigaku DMAX 2400, Japan) with a Ni filtered Cu Kα radiation was employed to identify the phase constitution of the experimental Ti‒2X alloys.

### Mechanical properties tests

The strip specimens (40 × 3 × 2 mm^3^) of as-cast binary Ti‒2X alloys were prepared for uniaxial tensile test, with pure Ti as control. The tensile test was performed with an initial strain rate of 5 × 10^−^ s^−1^ on a universal testing machine (Instron5969, USA) at room temperature. Five duplicate specimens were tested for each alloy. The Vickers microhardness of as-cast binary Ti‒2X alloys was measured on a digital microhardness tester (Shimadzu HMV-2T, Japan) at a load of 200 g for 15 s, repeating eight times in different positions for each alloy.

### Castability test

The castability of as-cast Ti‒2X alloys was conducted by modified Whitlock’s method^26^. with pure Ti as control. The details of this method can be found in our previous study^21^. A pure Ti casting system (SYMBION CAST, Nissin Dental Products INC., Japan) was used for casting the alloys and an argon pressure of 1.5 Pa was maintained during the casting. After casting, the castability value was obtained by using Whitlock’s formula, as shown in Eq. (1). Here the number of total wax mold segments equals 200, and the cast segment is deemed as complete or incomplete according to the previous work^26^.


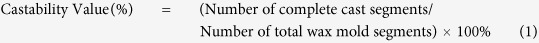


### Electrochemical measurements

The electrochemical measurements of as-cast binary Ti‒2X alloys were conducted on an electrochemical working station (Autolab, Metrohm, Switzerland) at 37 °C, with pure Ti as control. Two kinds of electrolytes were prepared from the analytic grade agents and de-ionized water. One is normal artificial saliva solution (AS, the composition can be seen in ref. 37), the other is extreme artificial saliva solution (ASFL, AS containing 0.2% NaF and 0.3% lactic acid, all additives were in weight percentage). The specimens with an exposed area of 1 cm^2^, in turn, were grounded, polished and ultrasonically washed. Three duplicate specimens were tested for each alloy. In the test, a platinum counter electrode and a saturated calomel electrode (SCE) reference electrode were used. The open-circuit potential (OCP) of each specimen was continuously monitored for 2 h in electrolytes. Afterward, the potentiodynamic polarization test was measured with a scan rate of 1 mV·s^−1^. Corrosion parameters including corrosion potential (*E*_corr_) and corrosion current density (*I*_corr_) can be estimated from the polarization curves by Tafel analysis based on the polarization plots. The electrochemical impedance spectroscopy (EIS) analysis has been performed in order to reveal the passive film formed on the surface of the samples. The EIS was acquired in the frequency range of 10^5^ Hz down to 10^−2^ Hz with a 10 mV amplitude sine wave at OCP. The EIS analyses, the equivalent circuit modelling and the corresponding elements values were calculated with Zview software.

### X-ray photoelectron spectroscopy (XPS) analysis

X-ray photoelectron spectroscopy (XPS) analysis (Axis Ultra, Kratos Analytical Ltd, UK) was performed to analyze the surface composition of the passive films after electrochemical tests. The test parameters are listed as follows: mono Al Ka (1486.6 eV) radiation at vacuum pressure of 10^−9^ bar, 15 kV, and 15 mA. The binding energy was calibrated using C1s hydrocarbon peak at 284.8 eV.

### Cell experiments

The cell experiments was carried out according to ISO 10993-5:2009^38^. Murine fibroblast cells (L929) and human osteoblast-like cells (MG63) were adopted to evaluate the cytotoxicity of as-cast binary Ti‒2X alloys by MTT assay. L929 and MG63 cells were cultured in Dulbecco’s modified eagle’s medium (DMEM) and minimum essential medium (MEM), respectively, in a humidified atmosphere with 5% CO_2_ at 37 °C. Both mediums were supplemented with 10% fetal bovine serum (FBS), 100 U·ml^−1^ penicillin and 100 μg·ml^−1^ streptomycin. Extracts were prepared using a serum-free medium as the extraction medium. The extraction ratio is 3 cm^2^/ml and the extraction was conducted for 72 h at 37 °C. Cell culture medium was used as a negative control and cell culture medium containing 10% dimethylsulfoxide (DMSO) as a positive control. Cells were seeded in 96-well plates at a density of 5 × 10^3^ cells per 100 μl medium and incubated for 24 h to allow attachment. Then the cell culture mediums were substituted by extracts, and incubated for 1, 2 and 4 days, respectively. After each culture period, 10 μl 3-(4,5-dimethylthiazol-2-yl)-2,5-diphenyltetrazolium bromide (MTT) was added into each well for 4 h incubation. Then 100 μl formazan solubilization solution (10% sodium dodecyl sulfate (SDS) in 0.01 M HCl) was added into each well overnight in the incubator. The spectrophotometric absorbance of the product in each well was measured with a microplate reader (Bio-RAD680) at 570 nm with a reference wavelength of 630 nm.

The alkaline phosphatase (ALP) activity was tested according to our previously reported protocols^22^. And the main steps are as below. MG63 cells were cultured in extracts for 7 days with an initial density of 5 × 10^3^ cells per 100 μl medium in 96-well plates. After 7 days’ culture, the culture medium in each well was removed and 100 μl of 1% Triton X-100 was added to obtain the cell lysates. ALP activity was determined based on the principle that ALP hydrolyzes the phenylphosphate to phenol and phosphate at pH value equals 10. And the phenol reacted with 4-aminoantipyrine in the presence of potassium ferricyanide, followed by forming a red-colored chinone compound, and this absorbance is proportional to the ALP activity. The reaction lasts for 15 min at 37 °C and the absorbance of the red-colored chinone compound products was measured at 545 nm using a microplate reader (Bio-RAD680)^22^.

The methods were carried out in accordance with the approved guidelines. All experimental protocols were approved by the Institutional Ethics Committee of Peking University. Written informed consent was obtained from all subjects.

### Statistical analysis

Statistical analysis was performed with SPSS 18.0 for Windows software (SPSS Inc., Chicago, USA). All data were statistically analyzed using one-way analysis of variance (ANOVA), followed by the Tukey post hoc tests. A *p*-value < 0.05 was considered statistically significant difference with pure Ti, as indicated by an asterisk (*) in relevant tables and figures.

## Additional Information

**How to cite this article**: Li, H. F. *et al*. Screening on binary Ti alloy with excellent mechanical property and castability for dental prosthesis application. *Sci. Rep.* **6**, 37428; doi: 10.1038/srep37428 (2016).

**Publisher’s note**: Springer Nature remains neutral with regard to jurisdictional claims in published maps and institutional affiliations.

## Supplementary Material

Supplementary Information
