# Supplementary material for: Screening on binary Ti alloy with excellent mechanical property and castability for dental prosthesis application
Source: Sci Rep. 2016 Nov 22;6:37428. doi: 10.1038/srep37428 (PMC5118711; doi:10.1038/srep37428)
Supplement: Supplementary Information [file srep37428-s1.doc]

**Supplementary Information**

**Screening on binary Ti alloy with excellent mechanical property and castability** **for** **dental prosthesis application**

H.F. Li, K.J. Qiu, W. Yuan, F.Y. Zhou, B.L. Wang, L. Li, Y.F. Zheng, Y.H. Liu

S1. Corrosion parameters of as-cast pure Ti and Ti–2X alloys obtained from electrochemical measurements in AS and ASFL solutions.

| Materials | OCP(V, *vs* SCE) | | *E*_corr_ (V, *vs* SCE) | | *I*_corr_ (A·cm^˗2^) | |
| --- | --- | --- | --- | --- | --- | --- |
|  | AS | ASFL | AS | ASFL | AS | ASFL |
| Pure Ti | -0.332(0.055) | -1.043(0.011) | -0.342(0.071) | -1.032(0.018) | 5.350(1.928)×10^˗7^ | 1.163(0.199)×10^˗4^ |
| Ti–2Ag | -0.185(0.066) * | -0.916(0.034) * | -0.228(0.051) | -0.896(0.027) * | 0.416(0.068)×10^˗7^ * | 0.691(0.165)×10^˗4^ * |
| Ti–2Bi | -0.129(0.042) * | -1.034(0.008) | -0.311(0.131) | -1.022(0.008) | 0.660(0.067)×10^˗7^ * | 1.091(0.018)×10^˗4^ |
| Ti–2Ga | -0.263(0.074) | -1.051(0.008) | -0.267(0.033) | -1.030(0.005) | 4.260(1.053)×10^˗7^ | 1.038(0.036)×10^˗4^ |
| Ti–2Ge | -0.298(0.031) | -0.934(0.032) * | -0.353(0.072) | -0.941(0.003) * | 0.755(0.149)×10^˗7^ * | 0.844(0.186)×10^˗4^ |
| Ti–2Hf | -0.248(0.039) | -1.057(0.017) | -0.254(0.016) | -1.050(0.003) | 1.403(0.304)×10^˗7^ * | 0.983(0.019)×10^˗4^ |
| Ti–2In | -0.310(0.020) | -1.056(0.004) | -0.259(0.077) | -1.061( 0.001) | 3.217(1.216)×10^˗7^ | 1.050(0.148)×10^˗4^ |
| Ti–2Mo | -0.217(0.029) | -1.009(0.010) * | -0.275(0.055) | -0.937(0.057) * | 0.851(0.159)×10^˗7^ * | 0.965(0.091)×10^˗4^ |
| Ti–2Nb | -0.317(0.035) | -1.013(0.012) * | -0.297(0.027) | -1.011(0.001) | 5.517(0.930)×10^˗7^ | 1.062(0.068)×10^˗4^ |
| Ti–2Sn | -0.314(0.018) | -1.006(0.018) * | -0.306(0.018) | -1.012(0.006) | 0.388(0.129)×10^˗7^ * | 1.049(0.051)×10^˗4^ |
| Ti–2Zr | -0.144(0.046) * | -1.006(0.030) | -0.194(0.039) * | -1.025(0.014) | 2.763(0.686)×10^˗7^ * | 1.097(0.001)×10^˗4^ |
| Note: values in parenthesis represent the standard error (hereinafter the same). * indicates the statistically significant difference (*p*<0.05) with respect to pure Ti.  S2. Chemical compositions of Ti‒2X alloys.   | Alloys | Ti‒  2Ag | Ti‒  2Bi | Ti‒  2Ga | Ti‒  2Ge | Ti‒  2Hf | Ti‒  2In | Ti‒  2Mo | Ti‒  2Nb | Ti‒  2Sn | Ti‒  2Zr | | --- | --- | --- | --- | --- | --- | --- | --- | --- | --- | --- | | X (wt.%) | 1.77  (0.11) | 1.99  (0.25) | 1.98  (0.06) | 2.13  (0.05) | 2.25  (0.05) | 1.79  (0.05) | 2.22  (0.02) | 2.11  (0.08) | 2.15  (0.06) | 1.94  (0.15) | | Note: values in parenthesis represent the standard error (hereinafter the same). | | | | | | | | | | | | | | | | | |

S3. Equivalent circuit, R_s_(Q_p_R_p_), used for fitting the experimental data.

S4. Values of the circuit parameters obtained using the equivalent electrical circuit

R_s_(Q_p_R_p_) by fitting the experimental results of EIS.

| Material | R_s_ (Ω·cm^2^) | Q_p_ (Ω^-1^·cm^-2^·s^n^) (10^-5^) | n | R_P_(Ω·cm^2^) (10^5^) |
| --- | --- | --- | --- | --- |
| Pure Ti | 263.5 | 2.956 | 0.9077 | 3.63 |
| Ti-2Ag | 198.4 | 2.261 | 0.9419 | 85.13 |
| Ti-2Bi | 190.2 | 2.788 | 0.9282 | 27.17 |
| Ti-2Sn | 234.5 | 2.359 | 0.9205 | 31.37 |


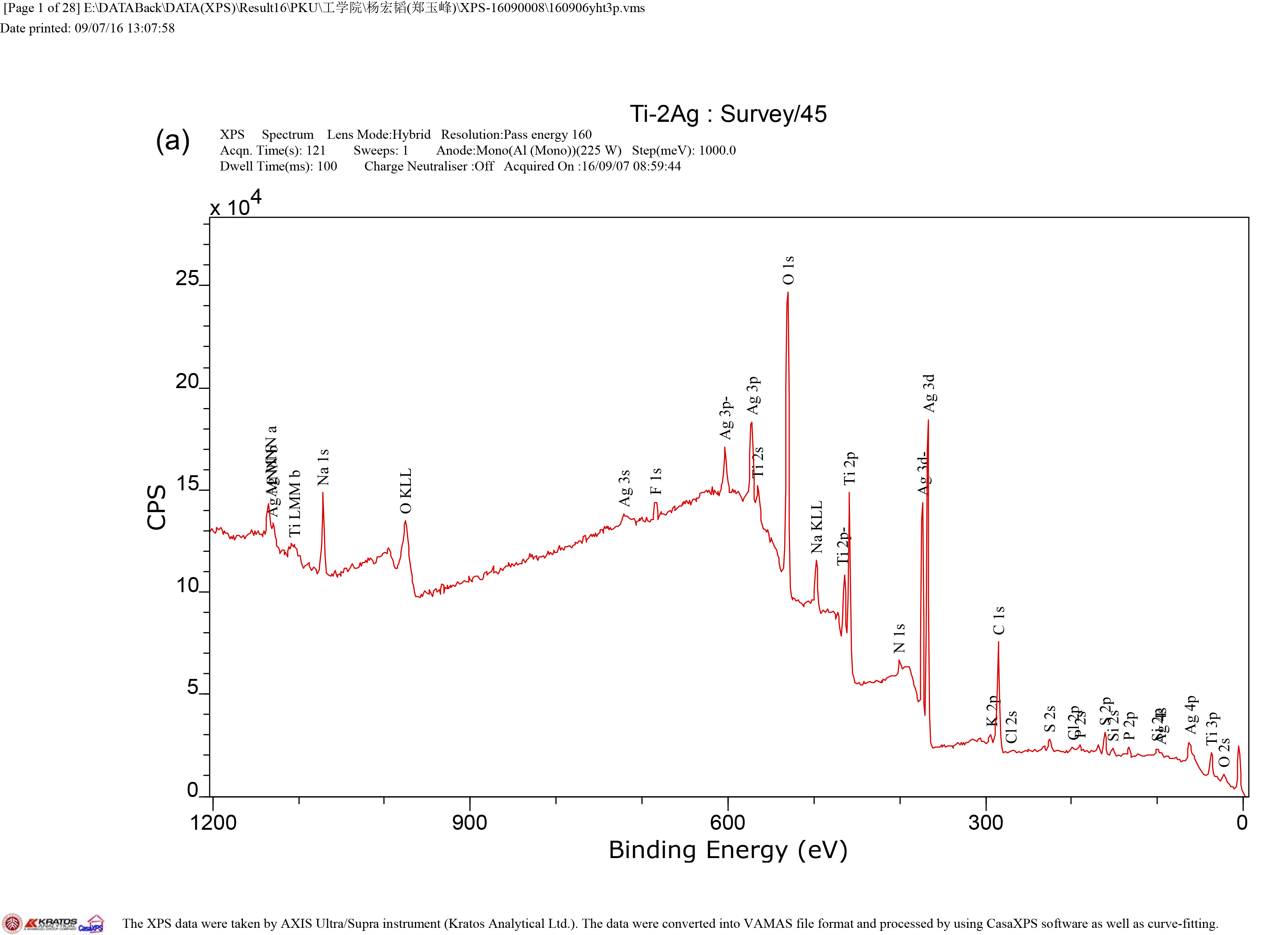


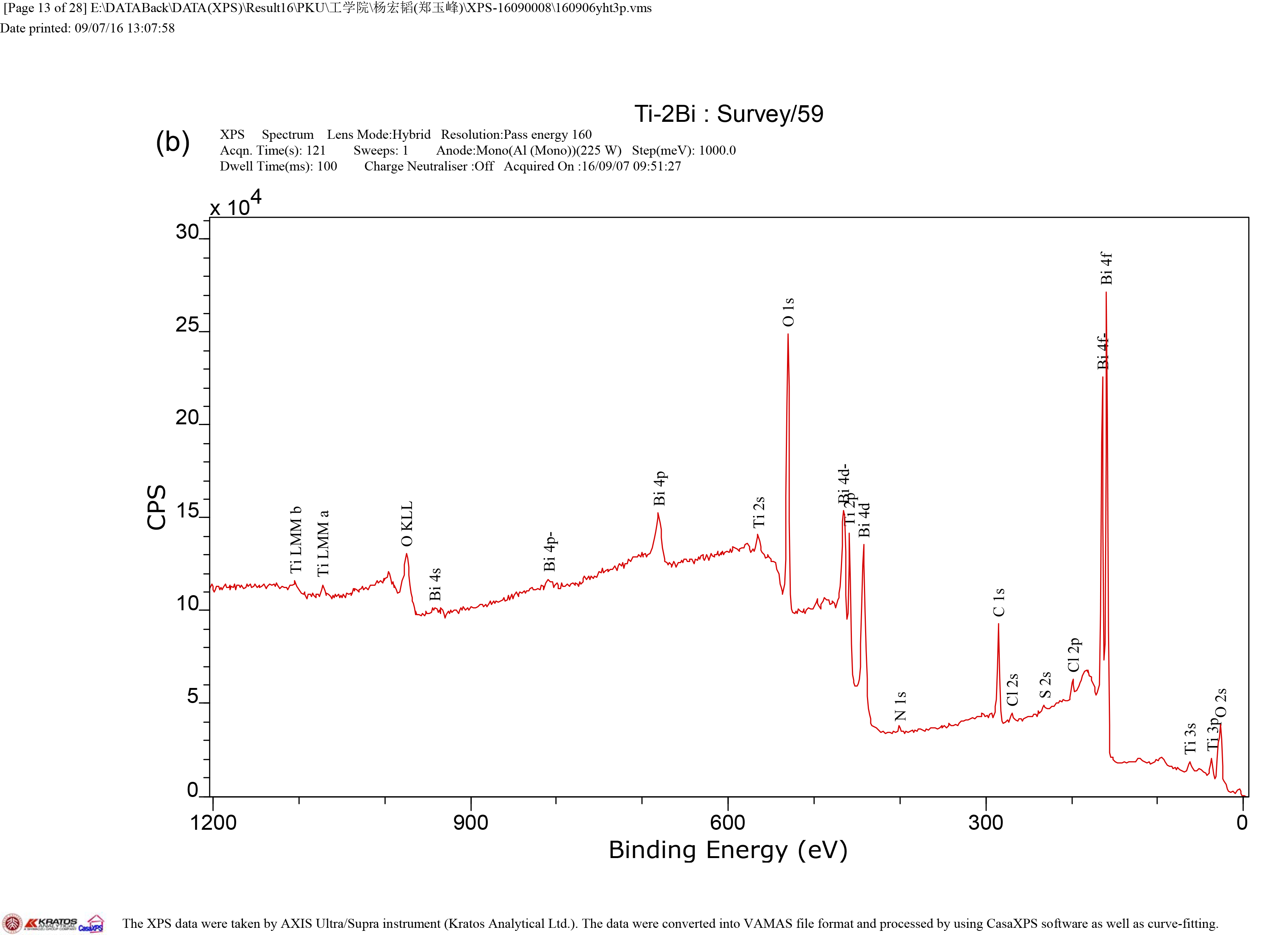


S5. XPS analysis results of the passive films after electrochemical tests (a) Ti-2Ag; (b) Ti-2Bi.
